# Case report of anti-NMDA receptor encephalitis in a 24-year-old female: an uncommon presentation

**DOI:** 10.1186/s41983-022-00512-7

**Published:** 2022-06-23

**Authors:** Antoine Steeman, Ionut Andriescu, Cécile Sporcq, Delphine Mathieu, Virginie Meurant, Guy Mazairac

**Affiliations:** 1 Emergency Department, CHU Tivoli, La Louvière, Belgium; 2 Emergency Department, CHR de Huy, Huy, Belgium; 3 Radiology Department, CHU Tivoli, La Louvière, Belgium; 4 Internal Medicine Department, CHU Tivoli, La Louvière, Belgium; 5 Emergency Department, St. Nikolaus Hospital Eupen, Eupen, Belgium

**Keywords:** Neurology, Psychosis, Autoimmune encephalitis, Confusion

## Abstract

**Background:**

Anti-N-methyl-d-aspartate receptor (NMDAR) encephalitis is a form of autoimmune encephalitis. Due to the variability of the initial symptoms, anti-NMDAR encephalitis is not only underdiagnosed but also can be misdiagnosed as viral encephalitis or other pathologies. The origin of this disease is often paraneoplastic. Anti-NMDAR encephalitis preferentially affects children and young adults, and it has a male/female ratio of 1/4. In case of clinical suspicion, electroencephalogram and brain magnetic resonance imaging are useful, but lumbar puncture for cerebrospinal fluid analysis is used to confirm the diagnosis. Treatment for this disease includes immunosuppression and tumour resection when indicated.

**Case presentation:**

We report the case of a 24-year-old female admitted to the emergency room following the onset of acute confusion. Due to the rapid deterioration of consciousness, swallowing disorders, respiratory failure and severe bradycardia the patient was intubated. On day 23 after presentation, brain magnetic resonance suggested autoimmune limbic encephalitis. Cerebrospinal fluid results were positive for anti-NMDA antibodies. After IV methylprednisolone and plasmapheresis and a second line therapy with corticosteroid and mycophenolic acid, the patient’s clinical condition gradually improved.

**Conclusions:**

Anti-NMDAR encephalitis typically occurs in young patients with no history of acute psychiatric symptoms. The possibility of this pathology should be taken into account before diagnosing a patient with a psychiatric illness.

## Background

Anti-N-methyl-d-aspartate (NMDA) receptor encephalitis is a rare autoimmune disease that is frequently underdiagnosed. The pathophysiology of this disease results from the binding of anti-NMDA antibodies to NMDA receptors, causing neuronal dysfunction and the disruption of fronto-striatal connections.

The clinical presentation of this disease is manifested by non-specific influenza symptoms, such as headache, fever, nausea and upper respiratory symptoms [1]. Subsequently, acute psychiatric symptoms appear, such as agitation, visual and auditory hallucinations, anxiety, emotional lability, catatonia and disorganized thoughts. Neurological deterioration typically occurs 1–3 weeks after the onset of symptoms, including abnormal movements, seizures and autonomic nervous system disorders with tachycardia, bradycardia, hyperhidrosis, hypersalivation, tension instability [2]. This clinical picture is often complicated by altered consciousness, swallowing disorders and respiratory distress, which requires intensive care.

## Case presentation

We report the case of a 24-year-old female patient with no significant history who was admitted to the emergency room following the onset of acute confusion and fear of imminent death. Few days before her admission, she had rhinitis with sore throat and headache.

In the emergency room, she presented agitation, hallucinations, echolalia with ideas of death and aggression. Physical and neurological examination were not showing pathological features. Laboratory testing demonstrated no abnormalities, with a normal white blood cell count and normal C-reactive protein. Toxicology screening for barbiturate, tricyclic, benzodiazepine, amphetamine, methamphetamine and cocaine was negative. Electroencephalogram (EEG) and brain scan were also normal. The patient was hospitalized for an additional assessment.

At first, there was a spontaneous resolution of her psychiatric symptoms. Two days later, the reappearance of agitation, impulsive aggression, and visual hallucinations led to psychiatric hospitalization on suspicion of anxiety disorder. In the psychiatric department, a treatment with olanzapine 30 mg per day, risperidone and lorazepam was initiated. Nevertheless, after 4 days, her clinical condition worsened, including impaired consciousness, pyrexia, tachycardia, swallowing disorders and food refusal. In views of this clinical picture, a neuroleptic malignant syndrome was suspected and the patient was transferred to the emergency room.

In the emergency room, she presented a state of catatonia with fever, tachycardia, tachypnea, muscle rigidity and swallowing disorders. Laboratory testing revealed inflammatory syndrome (CRP 50 mg/L) with a normal white blood cell count. Screening for autoimmune diseases (anti-neutrophil cytoplasmic antibodies, anti-nuclear antibodies) was negative. Lumbar puncture revealed transparent cerebrospinal fluid (CSF) with IgG–oligoclonal bands without pleocytosis and normal values for glucose and protein. Polymerase chain reactions for herpes zoster, enterovirus and herpes simplex on CSF were normal. Blood and CSF cultures were negative. Further immunologic tests on CSF were sent to a university laboratory. EEG and brain magnetic resonance imaging (MRI) findings were also normal.

**Fig. 1:**
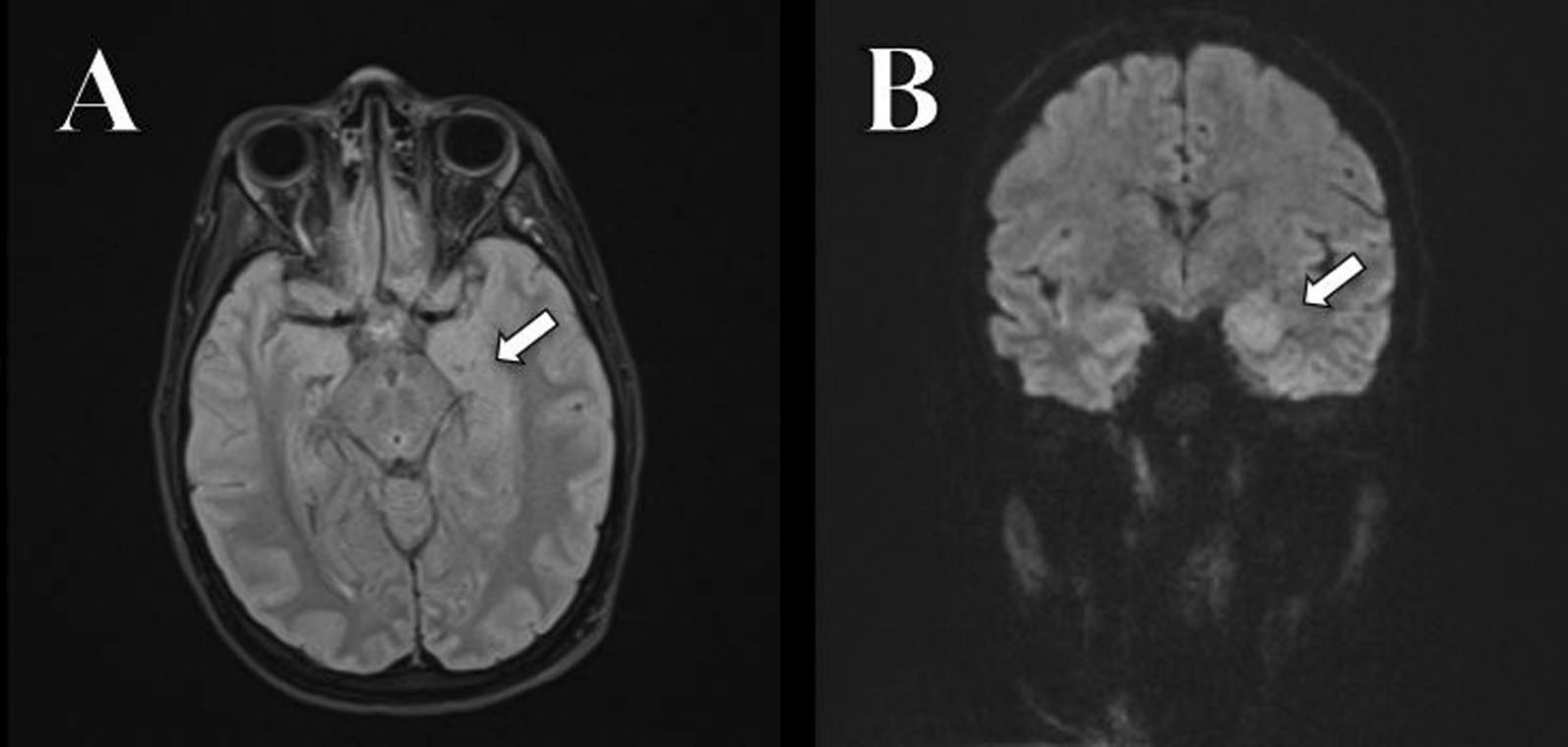
T2 axial FLAIR (**A**) and resolve 3 scan trace (**B**) showing asymmetry of hippocampal regions with a significant restriction to the left (arrows)

Due to the deterioration of consciousness, swallowing disorders, respiratory distress syndrome and severe bradycardia, the patient was intubated and transferred to the intensive care unit (ICU). Neuroleptics were stopped. On day 23, a new Brain MRI was performed and showed asymmetry of the hippocampal regions as well as a diffusion restriction on the whole temporal cortex (see Fig. 1), suggesting autoimmune limbic encephalitis. Pulsed therapy with 1 g/day methylprednisolone was initiated. The patient continued to develop dysautonomia disorders (low blood pressure, tachycardia, apnoea and Cheyne–Stokes respiration) as well as episodes of acute agitation. A tracheotomy was performed due to the necessity of a long-term mechanical ventilation. Finally, CSF results showed the presence of IgG oligoclonal bands and testing for anti NMDA receptor antibodies were positive in the CSF. The 48-h EEG did not demonstrate any epileptogenic activity. A transvaginal ultrasound and a whole-body PET–CT were performed in search of a tumoral origin, such as ovarian teratoma, which were both negative.

The patient benefited from intravenous methylprednisolone therapy as well as 10 plasmapheresis sessions, followed by oral corticosteroid therapy (64 mg/day as starting dose) followed by a mycophenolic acid 500 mg twice a day for 2 weeks then 1 g twice a day (to be continued for 6 months). The patient’s clinical condition gradually improved, and she was transferred to the neurology department.

A neuropsychological and neurological assessment showed a pyramidal syndrome with proximal predominant left hemiparesis, a deficit in verbal fluency and pathological denomination. Clinical progress was slow but favourable with corticosteroid therapy and mycophenolic acid treatment. The patient returned home after 56 days of hospitalization. At an 18-month follow-up, there was still a functional deficit (dexterity and balance) but the 18F-FDG PET scan for oncologic follow-up remained negative.

## Conclusions

Anti-NMDA receptor encephalitis preferentially affects children (from 2 months) and young adults (less than 5% of patients are over 45 years) with a male/female ratio of 1/4 [3]. The origin of this disease is often paraneoplastic. Approximately 50% of women over 18 years and only 9% of girls under 14 years have an ovarian teratoma [4]. In men, the presence of tumours is rare [5].

The diagnosis is confirmed by the detection of IgG antibodies directed against the GluN1 subunits of the NMDA receptors in serum and CSF [6] which is not available at the time of presentation to the emergency department. Therefore, when a patient presents to the emergency department with suggestive clinical picture, lumbar punction should be performed to look for CSF pleocytosis or oligoclonal band. EEG is useful and shows abnormal results in the majority of cases but nonspecific with a slow and disorganized epileptic activity [7]. Brain MRI is often normal. In the study of Dalmau and colleagues [7] only 55% of patients had increased FLAIR or T2 signal in the cortical or subcortical areas (hippocampus, basal ganglia, white matter).

The differential diagnosis includes acute primary psychiatric disorder, neuroleptic malignant syndrome, malignant catatonia, drug intoxications, viral encephalitis, and lethargic encephalitis [8].

However, the diagnosis of anti-NMDA receptor encephalitis remains difficult due to the vagueness of the primary clinical picture. In a study [9] anti-NMDA antibodies are found in 50% of patients diagnosed with lethargic dyskinetic encephalitis. In addition, 20–30% of patients with herpes simplex virus (HSV) infection show positive seroconversion with anti-NMDA antibodies as part of a relapse not attributable to HSV relapse [10].

In the absence of reliable statistical data, there is no standard treatment; instead, treatment should be individualized according to age, the severity of symptoms, and the presence or absence of a tumour. Treatment can include immunosuppression and tumour resection when indicated [4]. In the presence of CSF pleocytosis or oligoclonal band at lumbar punction and abnormal EEG or pathologic brain MRI, immunosuppressive treatment should be started.

Some therapeutic variants for the initial immunosuppressive treatment are recommended, including either an intravenous infusion of methylprednisolone (1 g/day for 5 days) or intravenous treatment with immunoglobulin G (400 mg/kg/day for 5 days) or plasmapheresis [11].

In the absence of clinical improvement, second-line treatment with rituximab (375 mg/m^2^/week for 4 weeks) or cyclophosphamide (750 mg/m^2^/month for 4–6 months) or a combination of both molecules can be proposed [11]. Mycophenolate mofetil can also be used as second line therapy. This drug has a selective antiproliferative activity on lymphocytes and has shown better efficacy in inducing remission [3]. Furthermore it has less side effect profile than cyclophosphamide in other autoimmune disorders [12]. Finally, for patients with severe and treatment-refractory disease, bortezomib [13] or tocilizumab [14] can be used as third-line therapy.

Although immunosuppression is the main treatment for NMDAR encephalitis, patients often require management in the emergency room. Benzodiazepines such as lorazepam are the first choice for treatment of catatonia [15]. For patients with agitation and hallucinations, atypical antipsychotics such as olanzapine are preferable, because they induce fewer side effects [3]. Valproic acid may be effective for mood disorders as well as dyskinesias and choreiform movements, which are common in anti-NMDAR encephalitis [16]. Antiepileptic drugs such as levetiracetam, phenobarbital, topiramate, lamotrigine, valproic acid, phenytoin and carbamazepine are recommended for the treatment of seizures. Patients with severe disease will require mechanical ventilation and intensive care management [2]. Due to cardiac autonomic dysfunctions, such as severe bradycardia, arrhythmia and hypotension, some patients will even require cardiopulmonary resuscitation and cardiac pacing.

In addition, patients may require long-term care in intensive care from several weeks to several months as well as multidisciplinary rehabilitation.

NMDA anti-receptor IgG crosses the placental barrier, and its effects on the foetus can be variable; case of early neonatal death has been described previously [3].

Even if this case happened before the COVID-19 pandemic, encephalopathy is common in critically ill patients with COVID-19 and it is related with increased mortality, independently from any respiratory disease severity. These patients typically have no evidence of brain inflammation on neuroimaging studies or on cerebrospinal fluid analysis. They were also more likely to be old, male, and with risk factors which is not the case presented in this study [17].

In conclusion, anti-NMDA receptor encephalitis is a rare disease underdiagnosed due to the variability of the initial symptoms. In general, emergency physicians are not familiar with this disease. In case of patient with suggestive clinical picture of anti NMDAR encephalitis presenting to the emergency room, lumbar puncture should be performed as soon as possible to look for CSF pleocytosis and oligoclonal bands. Abnormal EEG findings and brain MRI can help to not delay the initiation of immunosuppressive treatment. The diagnosis is confirmed by the detection of antibodies anti-NMDAR in the CSF. The possibility of this pathology should be taken into account as a differential diagnosis for patients presenting with acute psychosis or encephalitis before diagnosing a psychiatric illness.

## Data Availability

Data sharing is not applicable to this article as no data sets were generated or analysed during the current study.

## Abbreviations

NMDA: Anti-N-methyl-d-aspartate
NMDAR: Anti-N-methyl-d-aspartate receptor
CRP: C-reactive protein
EEG: Electroencephalogram
CSF: Cerebrospinal fluid
MRI: Magnetic resonance imaging
ICU: Intensive care unit
FLAIR: Fluid-attenuated inversion recovery
HSV: Herpes simplex virus
PET–CT: Positron emission tomography
IgG: Immunoglobulin G
18-FDG: Fluorodeoxyglucose
